# Morality on the road: the ADC model in low-stakes traffic vignettes

**DOI:** 10.3389/fpsyg.2025.1508763

**Published:** 2025-06-09

**Authors:** Michael Pflanzer, Dario Cecchini, Sam Cacace, Veljko Dubljević

**Affiliations:** ^1^Center for AI in Society and Ethics, North Carolina State University, Raleigh, NC, United States; ^2^Violence Prevention Center, University of North Carolina at Charlotte, Charlotte, NC, United States

**Keywords:** ADC model, autonomous vehicles, moral judgment, traffic morality, moral psychology

## Abstract

**Introduction:**

In recent years, the ethical implications of traffic decision-making, particularly in the context of autonomous vehicles (AVs), have garnered significant attention. While much of the existing research has focused on high-stakes moral dilemmas, such as those exemplified by the trolley problem, everyday traffic situations—characterized by mundane, low-stakes decisions—remain underexplored.

**Methods:**

This study addresses this gap by empirically investigating the applicability of the Agent-Deed-Consequences (ADC) model in the moral judgment of low-stakes traffic scenarios. Using a vignette approach, we surveyed professional philosophers to examine how their moral judgments are influenced by the character of the driver (Agent), their adherence to traffic rules (Deed), and the outcomes of their actions (Consequences).

**Results:**

Our findings support the primary hypothesis that each component of the ADC model significantly influences moral judgment, with positive valences in agents, deeds, and consequences leading to greater moral acceptability. We additionally explored whether participants’ normative ethical leanings–classified as deontological, utilitarian, or virtue ethics–influenced how they weighted ADC components. However, no moderating effects of moral preference were observed. The results also reveal interaction effects among some components, illustrating the complexity of moral reasoning in traffic situations.

**Discussion:**

The study’s implications are crucial for the ethical programming of AVs, suggesting that these systems should be designed to navigate not only high-stakes dilemmas but also the nuanced moral landscape of everyday driving. Our work creates a foundation for stakeholders to integrate human moral judgments into AV decision-making algorithms. Future research should build on these findings by including a more diverse range of participants and exploring the generalizability of the ADC model across different cultural contexts.

## 1 Introduction

Imagine driving during rush hour and navigating the morning traffic to get your child to middle school on time. As you approach an intersection, the traffic signal ahead switches to yellow. You are already running 10 min behind schedule, and the main road ahead appears deserted. What would you do? Traffic decision-making has received much attention in recent years following the deployment of automated vehicles (AV) (Lin, 2016; Awad et al., 2018). While there are many philosophical thought experiments for addressing ethical dilemmas and emergencies, traffic decision-making often occurs in mundane situations. Researchers have argued that automated driving systems must have the capacity to make traffic-related decisions that align with common sense moral judgment (Awad et al., 2018; Gil, 2021). As such, such intelligent systems should be programmed to act as moral agents capable of recognizing the most salient morally relevant factors. However, the main factors in traffic moral judgment and how they interact are still unclear and debated. This study aims to shed light on this problem.

Without understanding traffic moral judgments, there is a risk of inadvertently programming flawed or ethically contentious decision-making frameworks into AVs, potentially leading to unintended consequences and ethical dilemmas. Therefore, exploring the complexities of moral traffic behavior is not just about enhancing the safety and efficiency of AVs but also about aligning this emerging technology with common sense intuitions. This alignment is crucial for reassuring the audience about the ethical considerations in AV development. This approach necessitates interdisciplinary collaboration among ethicists, psychologists, engineers, and policymakers to develop robust frameworks reflecting moral decision-making nuances in traffic contexts. Our present research examines the moral preferences and judgments of experienced philosophers to contribute to a growing collective of disciplinary perspectives toward moral traffic behavior.

Published studies to date have primarily focused on examining traffic decisions in high-stakes emergencies, aligning with the influential “trolley problem” paradigm (Awad et al., 2018; Sütfeld et al., 2017; Faulhaber et al., 2019). The scenarios typically tested involve choosing between two unavoidable harms, such as swerving left and hitting an elderly person or proceeding forward and risking a child’s life as they cross the street. Trolley-like decisions offer a straightforward and replicable structure, making them valuable for understanding people’s consequentialist moral reasoning. However, criticism has been directed at the trolley paradigm for oversimplifying real-world traffic scenarios (Nyholm and Smids, 2016; Himmelreich, 2018) and lacking ecological validity (Bauman et al., 2014).

While high-stakes trolley-like situations have been extensively investigated, everyday low-stakes decisions like the one illustrated at the beginning of this section still need to be sufficiently studied. This constitutes a relevant research gap, considering that traffic conduct includes a variety of low-stakes moral scenarios such as intersection approaching, lane changing, or merging (Himmelreich, 2018; Borenstein et al., 2019). Situations like these are likely relevant to automated vehicle decision-making for multiple reasons. First, understanding how subjects respond to mundane situations may contribute to artificial agents developing a sensitivity to specific conditions based on contextual parameters, akin to human intuitive decision-making. Second, moral decision-making in everyday situations may contribute to the prevention of high-stakes emergencies, which do not arise without mundane bad decisions happening first. Therefore, studying everyday low-stakes traffic decisions would be a valuable contribution to automated vehicles’ decision-making.

Another neglected research theme thus far is how character-based considerations influence traffic moral judgment. Trolley-like dilemmas contrast deontological and consequentialist intuitions while overlooking a virtue-oriented viewpoint. Considering the documented significance of character information in moral psychology (Uhlmann et al., 2015), it is worthwhile to explore whether and to what extent displaying virtuous or vicious behavior in traffic influences moral judgment. For instance, it could be morally relevant to discern whether a particular vehicle is hastily transporting an injured person to the hospital or attempting to evade the police after a robbery. Such considerations capture the nuances of moral decision-making beyond simple comparisons between consequences and principles.

To address the limitations of the trolley paradigm and navigate moral complexity in traffic decision-making, some scholars have recently developed the Agent-Deed-Consequences (ADC) model of moral judgment (Cecchini et al., 2023; Dubljević, 2020). This framework, which has been applied to similar moral domains, posits that the moral acceptability of traffic conduct depends on positive or negative evaluations of three different components: the character and intentions of the driver (the Agent component, A), their compliance with traffic rules (the Deed component, D), and the outcome of the traffic maneuver (the Consequences component, C). The model predicts that the situation will be perceived as morally acceptable if all three A, D, and C components are deemed positive and unacceptable if all three components are viewed as negative. For example, suppose an observant ambulance driver (A+) drives carefully while transporting a severely burned patient to a hospital (D+), and, in the end, the patient is stabilized and recovering (C+). In that case, the moral judgment of the situation will be acceptable (positive moral judgment, MJ+). Conversely, if a terrorist (A–) uses his vehicle to ram pedestrians (D–) and several innocent people die (C–), the moral judgment will be unacceptable (negative moral judgment, MJ–). It becomes increasingly difficult to rate the acceptability of moral judgments when the valences of the A, D, and C components are incongruent. For example, if a drug dealer traveling with illicit materials (A–) attempts to save a bleeding child by carefully driving to the hospital (D+) and is ultimately unsuccessful as the child dies (C–), would the moral judgment of the situation be acceptable?

The primary theoretical advantage of the ADC model lies in its incorporation of character-based ethical considerations alongside consequentialist and deontological reasons (Aliman and Kester, 2019). This introduces greater complexity than the trolley paradigm. Nevertheless, the ADC model maintains a simple and repeatable structure, facilitating large-scale data collection and ease of programming into artificial agents. In this context, an algorithmic system could substitute an overall moral judgment with more accessible information in distinct computations (Pflanzer et al., 2023). Furthermore, given that the principal predictions of the ADC model have found support in various moral domains (Dubljević et al., 2018; Sattler et al., 2023), it is reasonable to expect that traffic-related moral judgments would follow similar trends. This study aims to test this hypothesis.

## 2 The current experiment

This study constitutes the first empirical investigation of the ADC model in the road traffic context, as some authors recently theorized (Dubljević, 2020). Therefore, the primary goal of the experiment is to ascertain whether traffic moral judgment is explained by the main hypotheses of the ADC model, in line with evidence in other domains (Dubljević et al., 2018; Sattler et al., 2023). In other words, we aim to test whether traffic moral judgment varies according to positive or negative evaluations of a driver’s character and intentions (the Agent component, A), their compliance with traffic rules (the Deed component, D), and the outcome of the traffic maneuver (the Consequences component, C). On this basis, Hypothesis One predicts that all three ADC components–Agent, Deed, and Consequence–will exert significant main effects on moral judgment. Drawing on prior research (Dubljević et al., 2018; Cecchini et al., 2023), we expect that positive agents, deeds, and consequences will result in higher moral judgment scores, and negative agents, deeds, and consequences will result in lower moral judgment scores. We anticipate that Deeds will have the most significant effect, followed by Agents and Consequences. However, these predictions remain tentative, given the novelty of applying the ADC model to low-stakes traffic scenarios.

Our use of the ADC framework draws from long-standing moral theories (Dubljević et al., 2018; Cacace et al., 2022; Cecchini and Dubljević, 2025), but similar integrative models have also emerged in recent years. For instance, Dyadic Morality (Schein and Gray, 2018) conceptualizes moral judgment as grounded in perceived harm, emphasizing the roles of both agents and victims within moral events. Similarly, Augmented Utilitarianism (Aliman and Kester, 2019) expands classical utilitarianism to include agent-based and contextual elements, acknowledging that moral evaluations often extend beyond outcomes alone, particularly for efforts to develop morally-aligned artificial intelligence. These frameworks underscore the growing recognition that moral judgment cannot be fully explained by a single normative theory and support our rationale for testing the interactive effects of agentic intent, moral action, and consequences. Nevertheless, it is worth noting that the ADC model has been more specifically tailored to the context of AV (Cecchini et al., 2023; Dubljević, 2020; Cecchini and Dubljević, 2025), as compared to other integrative approaches like Augmented Utilitarianism.

Interpretations of the components may vary according to individual differences, and the emphasis individuals place on specific components will likely determine the overall acceptability of moral judgment. Hypothesis Two predicts that preferences for an ethical standpoint (deontological, consequentialist, or virtue-oriented) will determine the weight assigned to each Agent, Deed, and Consequence component. Following previous research (Pflanzer et al., 2023), we theorize that subjects aligned with virtue ethics may assign more weight to agential intentions than to deeds and consequences. By contrast, information about the action outweighs agency and consequences in deontological intuitions. Finally, consequentialism prioritizes consequences over deeds, character, and intentions. Therefore, the current study aims to clarify the influence of the three major normative ethical frameworks on traffic moral judgment.

Finally, we predict interactions between the three ADC components. Specifically, we expect two-way interactions between Agent and Deed, Agent and Consequence, and Deed and Consequence to significantly influence Moral Judgment (MJ). While 3-way interactions (Agent × Deed × Consequence) are possible, they remain exploratory in this study and will be analyzed to detect potential patterns. This aspect is particularly relevant when moral components are not aligned. For instance, a car rushing to the hospital to transport an injured person (C+) may exceed the speed limit (D–), or a driver may exhibit kindness by allowing a dog to cross the street safely (A+), even if it involves stopping before a green light (D–). Hypothesis 3, therefore, tests for interaction effects between ADC components.

To test the hypotheses outlined above, we surveyed professional philosophers with at least a master’s degree in philosophy. The main rationale behind selecting this sample is the anticipation of consciously held and relatively well-defined preferences for moral theories among those with a philosophical background. Importantly, this choice facilitates the examination of the influences of virtue ethics, deontology, and consequentialism on moral judgment in particular situations. Although the reliability of philosophers’ moral judgments is currently a subject of debate (Schwitzgebel and Cushman, 2012; Horvath and Wiegmann, 2022), it remains reasonable to expect that individuals with philosophical expertise, alongside laypeople, contribute to informing the development of emerging technologies like automated vehicles (Savulescu et al., 2021). Thus, collecting data about philosophers’ traffic moral judgment might be helpful in the process of aligning driving automation systems with ethical standards. This study also represents a significant opportunity to enlarge the data sample of the ADC model, which thus far has been tested among laypeople but not professional philosophers.

As previously mentioned, existing literature has overlooked everyday traffic decision-making despite its significance as a crucial aspect of moral conduct on the road. To address this gap, the current experiment investigates professional philosophers’ moral responses to two low-stakes traffic scenarios. Indeed, the tested moral scenarios describe everyday driving tasks (e.g., bringing a child to school or a cat to the veterinary clinic), and the possible outcomes include minor incidents. As argued, we believe studying such mundane situations is necessary to integrate them into more dramatic life-and-death decisions.

## 3 Methods

### 3.1 Design and participants

We conducted a web-based vignette quasi-experiment characterized by an experimental approach without random selection (Shadish et al., 2002), in which we invited professional philosophers (those with at least a master’s degree in philosophy) from targeted university populations to complete a short (~15-min) Qualtrics® survey. Survey invitations were disseminated via the “PHILOS-L” mailing list and private emails to philosophy departments across North America (US and Canada). The emails were addressed to the heads of the targeted departments, who were asked to kindly share the survey with those affiliated with the department possessing at least a master’s degree in philosophy.

Of the 390 individuals who began the survey, we observed a 78.5% completion rate and a sample of 306 complete responses. During preliminary data analyses, linear response patterns were detected for 32 participants (10.5% of the sample). We defined a linear response pattern as participants selecting the same scale value (e.g., all 7 s or all 1 s) across all PPIMT or MJ items. While such uniformity could, in theory, reflect firm conviction, we excluded these cases based on the assumption that they were likely non-differentiated or careless responses (Leiner, 2019). Although the exclusion of these participants slightly decreased statistical power, we have nonetheless reported the statistical analyses conducted with the reduced sample size (*n* = 274; female = 80, male = 181, other = 13) as this approach improves the validity of our findings (Leiner, 2019).

This study was approved by an IRB committee (NCSU IRB No. 9484) and funded by the National Science Foundation CAREER award (#2043612.)

### 3.2 Materials and procedure

#### 3.2.1 Preference for precepts implied in moral theories

Moral preferences were measured using the Preference for Precepts Implied in Moral Theories (PPIMT) scale (Dubljević et al., 2022). The PPIMT scale was previously psychometrically validated, surveying a large sample of college students from a university in the southeastern United States and U. S. MTurk respondents (Dubljević et al., 2022). In the validation process, implicit moral preferences were tested to identify the most stable items characterizing the three major normative frameworks in ethics: Virtue Ethics (4 items), Deontology (4 items), and Utilitarianism (3 items) (see Table 1). This study utilizes a modified version of the PPIMT scale, which includes 11 rather than 15 items and was found to be superior to the full-scale (Dubljević, 2020; Dubljević et al., 2022).

**Table 1:** PPIMT prompts, organized by framework^a^.

PPIMT prompt	Mean	Standard deviation	PPIMT measures
*Virtue ethics (Agents)*	5.30	1.48	1, 11, 12, 15
Have good or bad intentions.	5.51	1.47	1
Have good or bad aims.	5.50	1.37	11
Have good or bad motives.	5.39	1.46	12
Have good or bad interests.	4.79	1.62	15
*Deontology (Deeds)*	4.75	1.58	7, 5, 10, 13
Respect or do not respect certain obligations.	5.27	1.44	7
Respect or do not respect certain rules.	4.36	1.71	5
Respect or do not respect certain norms.	4.30	1.70	10
Respect or do not respect certain duties.	5.05	1.47	13
*Utilitarianism (Consequences)*	5.45	1.36	6, 8, 14
Cause happiness or suffering.	5.83	1.17	6
Make somebody better or worse off.	5.44	1.40	8
Cause pleasure or pain.	5.07	1.51	14

^a^ Participants were asked to indicate their agreement with a phrase that begins with “When thinking about what is moral or immoral in a situation, it is important to me whether the involved persons” and ends with the PPIMT prompt reported in the table (7-point Likert scale: 1 = Disagree very much, 7 = Agree very much).

All PPIMT measures begin with the phrase, “When thinking about what is moral or immoral in a situation, it is important to me whether the involved persons….” Participants were then asked to rate their agreement on a range from “disagree very much” [1] to “agree very much” [7], with the remaining clauses that pertain to agents (virtue ethics), deeds (deontology), and consequences (utilitarianism). In the current sample, the estimated reliability (*α*) of the PPIMT was 0.635. To classify participants into ethical frameworks based on their PPIMT responses, we conducted K-Means Clustering, a technique that identifies groups of participants with similar response patterns. We specified three clusters to align with the theoretical frameworks of Virtue Ethics, Deontology, and Utilitarianism. Each participant was assigned to one of the three clusters based on the similarity of their responses to the cluster centroids. Framework assignments were validated by comparing the cluster means for the three PPIMT dimensions (Agent, Deed, and Consequence), which showed that the resulting groups closely corresponded to the expected ethical frameworks (Table 2). These cluster-based classifications were used in subsequent analyses to explore differences in moral judgment. The 3-cluster solution demonstrated a good fit (*R*^2^ = 0.69, Pseudo-*F* = 58.87, *p* < 0.001) and a high Cubic Clustering Criterion (CCC = 4.21).

**Table 2:** Frameworks were determined using K-Means Clustering, which grouped participants based on their response patterns to the PPIMT items into three distinct clusters corresponding to virtue ethics, deontology, and utilitarianism.

Framework	*n* (%)	PPIMT measure	Mean
Virtue Ethics	159 (58.0%)	Virtue ethics	6.02
		Deontology	5.47
		Utilitarianism	5.93
Deontology	37 (13.5%)	Virtue ethics	4.78
		Deontology	5.15
		Utilitarianism	3.75
Utilitarianism	78 (28.5%)	Virtue ethics	4.37
		Deontology	3.60
		Utilitarianism	5.53

#### 3.2.2 Moral judgment of traffic vignettes

We employed a 2 (positive vs. negative agent) × 2 (positive vs. negative deed) × 2 (positive vs. negative consequence) factorial survey design that merged the strengths of experimental approaches with those of survey-based approaches (Dülmer, 2016; Alexander and Becker, 1978). Simply put, the strength of the vignette design is that it more closely resembles real-life traffic scenarios (for greater external validity) while relying on quantifiable data for analyses (for greater internal validity). We created two low-stakes traffic Scenarios to demonstrate that the ADC model’s predictions are robust across different situations. Each scenario contains eight different vignettes corresponding to valence combinations for the various moral components. For example, a Vignette in which the Agent, Deed, and Consequence all had a positive valence would be coded as A + D + C +.

A vignette is a unique textual narrative of some traffic-related stream of events. The structure of each vignette is as follows: a protagonist driver is described as either virtuous or vicious (A+/A−). The driver then obeys or disobeys some traffic rule (D+/D−), which results in positive or negative consequences (C+/C−). For example, in the Intersection scenario, the protagonist driver is either a caring (A+) or abusive (A–) parent in a rush to drive their child to school; then, the parent encounters a yellow traffic light and either obeys the law and stops the car (D+) or quickly accelerates, intending to avoid the red light (D–); in the end, the child either arrives on time at school (C+) or the car is involved in a minor incident, and the child misses school (C–). In the Pet scenario, a son, described as being either compassionate (A+) or resentful (A−) towards a stray dog, is driving his disabled mother’s cat to the animal hospital when he either obeys traffic rules (D+) or ignores a stop sign (D−) and either safely delivers the cat to its appointment (C+) or causes an accident (C−). Each participant was randomly assigned to two vignettes, one for each scenario (Pet and Intersection). The two vignettes’ presentation order was also randomized to mitigate contrast, anchoring, and update effects.

**Figure 1:**
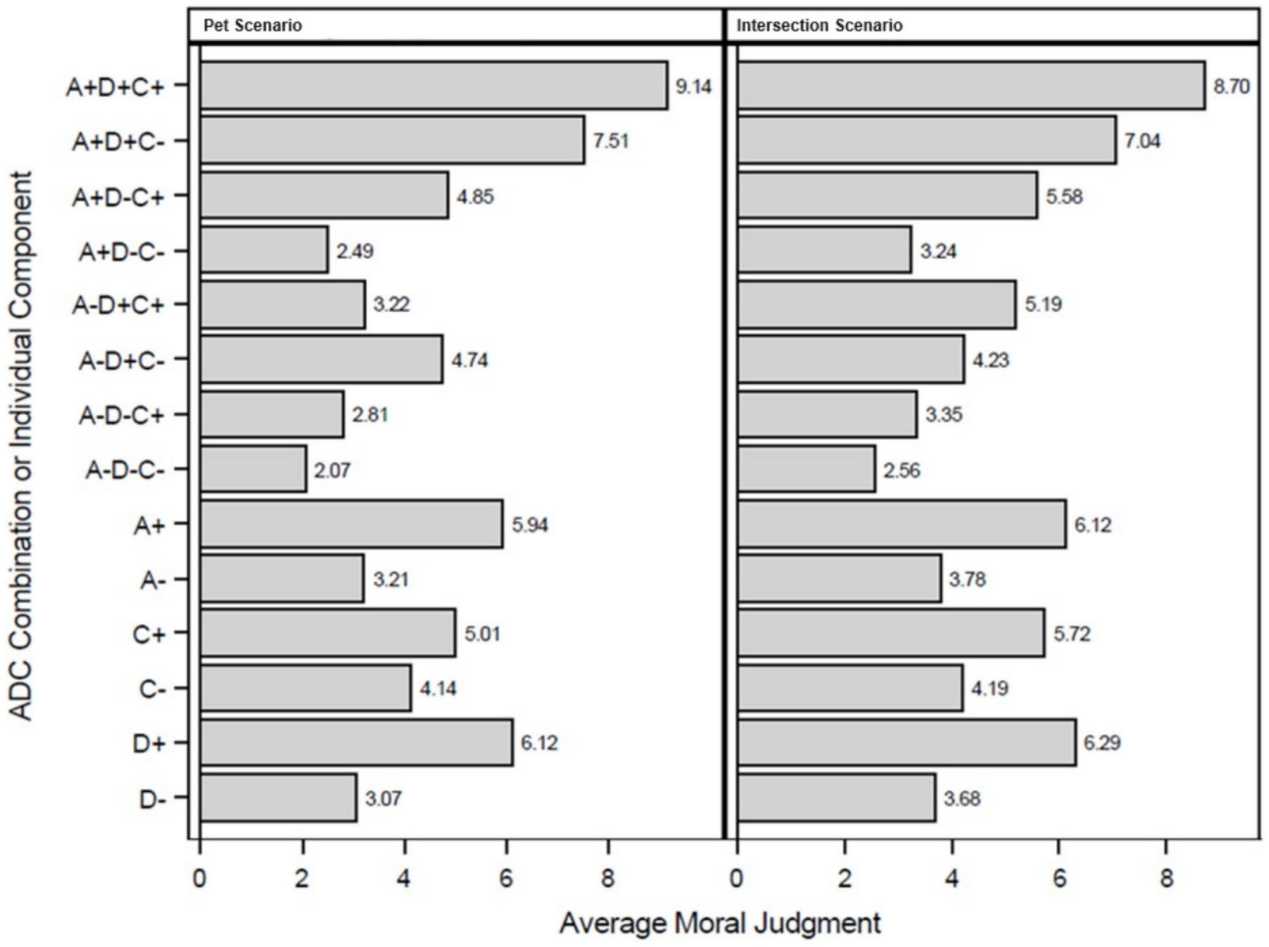
Average moral judgment. Average moral judgments are the calculated means of social and personal moral judgment.

After reading each vignette, participants were asked to consider all circumstances of the narrative and to rate both the *personal* and *social* moral acceptability of what the agent did in the situation (e.g., “for [(you personally) or (society)], how morally acceptable is what the man did in the situation?”). Response options varied from “not at all acceptable” [1] to “completely acceptable” [10]. We asked for both ratings because people sometimes have different expectations for personal and society moral behavior. During preliminary analyses of this data, however, the *personal* and *social* moral judgments were found to be highly correlated [*R*^2^ = 0.825, *p* < 0.0001], indicating convergence (in line with Dubljević et al., 2018), and thus, the means of personal and social moral acceptability were used to reflect moral judgment. Moral judgment, therefore, represents the mean moral acceptability, and this measure was calculated for all vignettes (see Figure 1).

### 3.3 Statistical analysis

Three models were tested using Analysis of Variance (ANOVA). Model 1 examines differences in Moral Preference and the Agent, Deed, and Consequence valences of each Vignette on Moral Judgment. Repeated measures were used to account for each participant’s observation of two different vignettes (one for the Pet and Intersection Scenarios).

To test the relative influence of Agent, Deed, and Consequence information on moral judgment, we assigned valence-based scores to each of the three moral components (A, D, and C) based on the structure of each vignette (e.g., a value of 1 for “positive,” and-1 for “negative).” The scoring rubric was applied uniformly across both Dog and Intersection scenario types. These ADC component scores were then entered as fixed effects in a mixed-effects model to assess their main and interactive contributions to participants’ moral judgment ratings. Model 2 examines the relationship between Framework and these ADC component scores on Moral Judgments. Type III errors were utilized to improve statistical validity when examining statistical significance due to unequal sampling of participants according to the Framework. This method accommodates unbalanced data by avoiding the sequential analyses of factors inherent in Type I testing and assuming the orthogonality of parameter additions (Zimmerman and Zimmerman, 2020). Model 3 examines the main effects of Scenario and ADC components and the interaction effects between ADC components. Contrasts were then later performed using the ESTIMATE function of GLM to conduct Tukey HSD *post hoc* analyses of the main effects of vignettes and scenarios and interactions between framework and vignette. Statistical analyses were conducted using SAS v. 9.4.

### 3.4 Scenario valence validity

Type III analysis of variance testing was employed to validate the expected variance in Moral Judgment across Scenarios (Pet or Intersection) and Vignettes (e.g., 1 = A+, C+, D+, 2 = A+, D+, C–, etc.). Moral Judgment was found to vary significantly across both Scenarios (*F*_1, 258_ = 5.28, *p* = 0.022) and Vignettes (*F*_7, 258_ = 32.57, *p* < 0.0001). Vignettes were further validated by contrasting each set of component valences (e.g., positive deeds with negative deeds, etc.) while controlling for the variances of the other two components. All three pairwise comparisons revealed statistically significant differences in Moral Judgment values: ΔMJ_A_ (*t* = 7.62, *p* < 0.0001, *σ* = 0.272), ΔMJ_D_ = 2.73/. (*t* = 10.07, *p* < 0.0001, σ = 0.272), and ΔMJ_C_ = 1.08 (*t* = 3.97, *p* < 0.0001, σ = 0.272). These comparisons reveal that of the three ADC components, the valence of the Deed component corresponded to the greatest difference in Moral Judgment scores (i.e., the MJ for vignettes in which the Deed was positive was, on average, 2.73 points higher than vignettes in which the Deed was negative).

## 4 Results

The primary objective of this study was to evaluate the influence of Agent, Deed, and Consequence (ADC) components (see Figure 1) on moral judgment (MJ) while accounting for individual differences in moral preferences (ethical frameworks: Deontology, Utilitarianism, and Virtue Ethics). Three hypotheses were tested using a combination of mixed-effects models, ANOVA, and *post hoc* comparisons, followed by robustness checks to validate the findings.

- H1: The valence of ADC components (Agent, Deed, and Consequence) will exert significant main effects on Moral Judgment across scenarios.
- H2: Preferences for a specific ethical framework (Deontology, Utilitarianism, or Virtue Ethics) will moderate the relative influence of ADC components on Moral Judgment.
- H3: Significant two-way and three-way interactions will occur between the ADC components, influencing Moral Judgment.

### 4.1 Main effects of ADC components (H1)

A repeated-measures linear mixed model was conducted to test the main effects of Agent, Deed, and Consequence on moral judgment. Significant main effects were found for each component:

- Agent: *F*(1, 266) = 15.25, *p* < 0.0001, ω^2^ = 0.051
- Deed: *F*(1, 266) = 29.85, *p* < 0.0001, ω^2^ = 0.098
- Consequence: *F*(1, 266) = 11.45, *p* = 0.0008, ω^2^ = 0.038

These results support Hypothesis 1, demonstrating that each ADC component independently influences participants’ moral evaluations. The reported Cohen’s ω^2^ values represent the proportion of variance in moral judgment attributable to each effect, with values above 0.01 generally considered small, 0.06 medium, and 0.14 large effects.

### 4.2 Moderation by ethical framework (H2)

A 2 × 2 × 2 × 3 mixed-effects factorial ANOVA tested whether moral preferences moderate the effects of ADC components. The main effects of Agent (*p* = 0.0131), Deed (*p* < 0.001), and Consequence (*p* = 0.0119) remained statistically significant. However, no interaction between Framework and any ADC component was statistically significant. To further explore this null effect, we conducted individual-level regressions for each participant and compared the resulting Agent, Deed, and Consequence weights across groups. No statistically significant differences emerged. Thus, Hypothesis 2 was not supported.

### 4.3 Interaction effects (H3)

Significant interactions emerged between ADC components and the scenario (Intersection vs. Dog):

- Agent × Scenario: *F*(1, 266) = 41.82, *p* < 0.0001, ω^2^ = 0.132
- Deed × Scenario: *F*(1, 266) = 29.69, *p* < 0.0001, ω^2^ = 0.097
- Consequence × Scenario: *F*(1, 266) = 7.78, *p* = 0.0057, ω^2^ = 0.025
- Agent × Deed × Scenario: *F*(1, 266) = 4.85, *p* = 0.0285, ω^2^ = 0.014
- Agent × Consequence: *F*(1, 259) = 6.54, *p* = 0.009, ω^2^ = 0.022

Other interactions were not significant. These findings partially support Hypothesis 3, indicating that the impact of ADC elements varies across contexts and component combinations. Pairwise comparisons between Agent and Consequence conditions revealed statistically significant differences in all combinations (see Table 3).

**Table 3:** Pairwise comparisons of agent and consequence valences.

Agent	Consequence	Estimate	Std Error	Pr > |t|
Negative	Negative	3.400	0.301	<0.0001
Negative	Positive	4.161	0.320	<0.0001
Positive	Negative	5.094	0.303	<0.0001
Positive	Positive	7.169	0.293	<0.0001

### 4.4 Scenario-specific analyses

To assess the consistency of moral judgment effects across contexts, we conducted separate 4-way ANOVAs for the Pet (Dog) and Intersection scenarios:

- Dog Scenario: *F*(23, 249) = 0.98, *p* = 0.49, *R*^2^ ≈ 0.08 (poor fit)
- Intersection Scenario: *F*(23, 251) = 10.43, *p* < 0.001, *R*^2^ ≈ 0.49 (strong fit)

Because the Dog scenario accounted for minimal variance and yielded non-significant results, it was excluded from subsequent hypothesis testing. The Intersection scenario alone was next used to re-evaluate the robustness of our findings.

### 4.5 Hypotheses re-tested using intersection data

Given the poor model fit observed in the Dog scenario, we conducted exploratory re-analyses using only the data from the Intersection scenario, which demonstrated a stronger model fit and explained substantially more variance in moral judgment. This allowed us to assess the robustness and generalizability of our findings across scenario contexts.

- H1 was again supported: Agent [*F*(1, 251) = 86.85, *p* < 0.001, ω^2^ = 0.15], Deed [*F*(1, 251) = 94.21, *p* < 0.001, ω^2^ = 0.12], and Consequence [*F*(1, 251) = 32.97, *p* < 0.001, ω^2^ = 0.04] all significantly predicted moral judgment, replicating the overall pattern observed in the full-sample model. The effect sizes for these analyses are higher than the combined Scenario analysis.
- H2 remained unsupported: no significant interactions between Framework and ADC components were observed. All Framework and ADC component interactions failed to reach statistical significance (*p*s > 0.05), and the effect size estimates were negligible (ω^2^ ≈ 0), suggesting that moral preferences did not meaningfully moderate how participants evaluated Agent, Deed, or Consequence in the Intersection context.
- H3 was further supported. Significant interactions were found between Agent and Consequence [*F*(1, 251) = 8.56, *p* = 0.0038, ω^2^ = 0.02] and Agent and Deed [*F*(1, 251) = 7.79, *p* = 0.0056, ω^2^ = 0.01], indicating that the influence of the valence of some components of MJ depended on the valence of another. Tables 3, 4 demonstrate the mean differences in MJ and the statistical significance of pairwise comparisons of these components.

**Table 4:** Pairwise comparisons of agent and deed valences.

Agent	Deed	Estimate	Std error	Pr > |t|
Negative	Negative	2.873	0.280	<0.0001
Negative	Positive	4.669	0.284	<0.0001
Positive	Negative	4.590	0.270	<0.0001
Positive	Positive	7.768	0.272	<0.0001

## 5 Discussion

While our testing scenarios do not include references to AVs, we believe that examining the way humans judge the moral acceptability of traffic-related decision-making is a necessary precursor to programming AVs to behave in ways society will find morally acceptable. This research seeks to pioneer methodologies for understanding moral judgment of traffic behavior. The present study only examines the moral evaluations of professional philosophers. Still, future studies that apply our methodology to other populations (e.g., taxi drivers, police officers, students, etc.) will contribute to a greater understanding of how humanity expects self-driving cars to behave, providing a foundation upon which they can later be imbued with moral agency. Our results supported the most important prediction of the ADC model. With a positive valence as compared to a negative valence of Agent (e.g., a caring parent vs. a negligent or abusive one), the Deed (e.g., obeying traffic rules vs. passing illegally), and the Consequence (e.g., arriving to school on time vs. missing school due to a traffic accident), the situations were judged as being significantly more acceptable. This is the first direct empirical corroboration of the ADC model in traffic situations. Thus, it serves as a ‘proof of principle’ that will be useful in assessing the moral acceptability of traffic-related behavior.

Interestingly, Hypothesis 2, which predicted that ethical frameworks would significantly influence the weights participants implicity assign to ADC components when forming moral judgments, was not supported. Instead, we observed more variation within frameworks than between them. This suggests that moral judgments in traffic scenarios may exhibit a high degree of consistency, even among professional philosophers with differing theoretical commitments. Rather than aligning strictly with their respective ethical doctrines, participants appeared to rely on shared intuitive responses, pointing to a potential universal core in moral evaluation within this domain. Although this finding may seem to challenge the expected influence of ethical frameworks, it carries important implications. The lack of divergence among philosophical perspectives strengthens the case for broad societal agreement on moral intuitions in traffic dilemmas, supporting the ADC model as a robust explanatory framework. If moral intuitions about traffic behavior remain stable across competing ethical traditions, this increases the likelihood of developing widely accepted ethical guidelines for AVs. Such convergence offers a promising foundation for establishing normative principles that align with public moral expectations, ultimately facilitating the integration of AVs into society.

Our findings indicate that the effects of a positive consequence (C+) on moral judgment were stronger for positive agents (A+) than for negative agents (A–). This may be an artifact of an expectation that the valence of agents, deeds, and consequences are congruent (e.g., “good behavior correlates with good consequences”) or that it is difficult to judge the moral acceptability of incongruent valences (e.g., “why did the good agent suffer bad consequences?”). Also, if Agent was positive rather than negative, the description of a positive Consequence (i.e., that the protagonists are out of danger) had a more substantial positive effect. We also found that the effects of negative consequences were stronger for negative deeds than for positive deeds. This implies that situations were judged to be more morally acceptable if the agent had good intentions and behaved morally. If such findings are replicated in future studies, we may theorize that it is more critical for humans and artificial agents to follow the rules than break them to avoid low-stakes adverse outcomes. This aligns with the common belief that negative deeds are associated with negative consequences.

### 5.1 Limitations and future improvements

Although our study provides preliminary evidence for the ADC model in traffic, it has some relevant limitations. One of the main issues is that the protagonists of the scenarios are drivers with human traits rather than AV. Future studies should evaluate if the moral judgment of AV behavior corresponds to the core principles of the ADC model. For this purpose, the challenge we foresee is translating human character (A), deeds (D), and consequences into algorithmic decision-making. To facilitate the transition from moral judgment in traffic scenarios to AV decision-making, ethical settings should be designed using numerical values (ranging from 0 to 1) for each of the three components of traffic conduct. This approach should consider not only the valence of each component but also its relative weight, as emphasized in previous theoretical work on the ADC model (Pflanzer et al., 2023; Cecchini and Dubljević, 2025).

Another important limitation is that moral scenarios employed in this study display *actual* consequences rather than the risk of collision. Since AV decision-making should be based on risk calculated before accidents occur rather than after, respondents should not be influenced by what happens to a vehicle, but they should judge each decision only according to the *expected consequences*. Displaying actual consequences exposes moral judgment to the notable phenomenon of *moral luck* (Kneer and Machery, 2019; Kneer and Skoczen, 2023). This means that respondents may consider unlucky drivers, whose decisions result in bad outcomes due to unfortunate circumstances, as more negligent (or reckless) than lucky agents making the same risky decisions. In other words, evaluating an action’s consequences (C component) might affect how an agent is evaluated (A component).

Furthermore, it is noteworthy that our study tests moral judgment and not decision-making. Indeed, previous studies on traffic culpability suggest that third-person judgments are subject to fundamental attribution error (Cacace et al., 2022). Research suggests, for example, that actors tend to attribute variations in performance to task difficulty, while observers attribute such variations to the actor’s ability (Jones and Nisbett, 1987). More precisely, people tend to underestimate the circumstances in which an accident occurs when assessing a stranger’s driving conduct. While we recognize that there are limitations of 3rd person vignettes (Flick and Schweitzer, 2021), the public perception of AV actions is necessarily comprised of moral judgments. Third-person moral judgments were selected for this study because they closely resemble how AVs will be evaluated by third parties—such as other road users, the public, and regulatory bodies—in real-world traffic situations. Given that AVs do not possess subjective experiences or first-person perspectives, understanding how external observers morally evaluate AV behavior is crucial for aligning AV decision-making with societal moral expectations.

Progress in understanding traffic-related moral decision-making can be made by using, for example, a virtual driving simulator, already in use in some studies investigating traffic decision-making (Faulhaber et al., 2019). In a first-person experimental setup, participants could take on the role of drivers making morally significant decisions in traffic, allowing researchers to assess whether their choices align with the ADC model. Alternatively, participants could experience being passengers in AVs programmed with ethical settings based on the ADC model while experimenters measure their moral judgments and trust in the technology. These empirical insights would further validate the application of the ADC model to traffic ethics, facilitating the transition from a third-person theoretical framework to a first-person experiential perspective. Adopting an immersive environment like a driving simulator would also address some limitations inherent in textual vignettes, such as ecological validity (Cecchini et al., 2023). Prior research has demonstrated that text-based scenarios tend to elicit more deontological responses compared to immersive virtual reality (VR) paradigms, which can increase the likelihood of utilitarian action (Francis et al., 2016; Maćkiewicz et al., 2023). These modality-dependent differences raise questions about the generalizability of our findings to real-world moral decision-making. Future work could benefit from comparing results across narrative, behavioral, and virtual modalities to further validate the observed patterns in moral judgment.

While this study utilized two distinct moral scenarios to assess generalizability, *post hoc* analyses revealed that the Pet Scenario demonstrated poor model fit and weak predictive power relative to the Intersection Scenario. The Pet Scenario accounted for minimal variance in moral judgment scores and did not yield significant effects, suggesting it may not have effectively manipulated the targeted moral dimensions (Agent, Deed, and Consequence). As a result, interpretations based on the Pet Scenario should be treated with caution, and future studies should refine or replace underperforming scenarios to ensure more robust measurement across moral contexts. Additionally, expanding the diversity of the sample beyond professional philosophers could also reduce the impact of sample-specific issues and improve the generalizability of the results. A more diverse sample, for example, should include laypeople, professionals from other fields (e.g., public transit drivers, policymakers), and individuals from different cultural contexts to improve the generalizability of the findings.

Previous research has incorporated low and high-stakes consequences in an experiment featuring ADC vignettes and found that the moderating effects of A, D, and C on moral judgment were different for low-stakes than for high-stakes scenarios (Dubljević et al., 2018). Although most human and autonomous drivers’ decisions occur in mundane situations with low-stakes consequences, operating motor vehicles will always entail some degree of risk for high-stakes consequences. Incorporating low and high-stakes consequences in future moral traffic research will provide essential nuance that extends the generalizability of a morally acceptable traffic decision-making framework.

Other research uses more complex models to analyze the mediation, moderation, and moderated mediation effects between components, moral judgment, and behavioral willingness to cooperate (Sattler et al., 2023). After accounting for linear and incomplete responses, we lacked a sufficient sample size to model the complex interaction effects between variables similarly. Future research will benefit from a more robust sampling method that either improves the response rate and diminishes the likelihood of linear responses or improves the sample size. However, this may be difficult as we also recommend assigning participants to more vignettes [following previous work (Dülmer, 2016), which proposes that vignette studies conducted this way have much stronger internal and external validity].

Another limitation is that this study was done in English with a sample of primarily North American respondents. Similar studies conducted in low-to-middle-income countries and in different languages need to be conducted to test the generalizability of these findings. While our initial study focused on philosophers to assess theoretical robustness, future research aims to expand the participant base to include traffic experts and laypeople, aligning with recent calls to improve ecological validity in moral judgment research.

## 6 Conclusion

This study explored the moral judgments of low-stakes traffic scenarios using the ADC model, which integrates character-based, deontological, and consequentialist considerations. Our findings indicate that each component of the ADC model significantly influences moral judgment, with positive valences in agents, deeds, and consequences leading to higher moral acceptability. This supports the hypothesis that these components collectively shape moral evaluations in traffic contexts.

The study’s results contribute to a better understanding of human moral reasoning to inform the development of ethical AVs. By empirically validating the ADC model in mundane traffic scenarios, we have laid the groundwork for integrating nuanced human ethical judgments into AV decision-making algorithms. The insights gained from this research underscore the importance of programming AVs to recognize and respond to the moral complexities of everyday driving beyond high-stakes emergencies typically examined in thought experiments such as trolley problem paradigms.

Furthermore, our study reveals that moral judgments are influenced by the interplay of agent character, rule adherence, and outcomes, with notable interaction effects. These findings highlight the intricate nature of moral reasoning and the potential challenges in replicating such judgments within AV systems. Our approach, which includes surveying professional philosophers, provides a robust methodology for capturing detailed ethical perspectives that can enhance AV programming.

Future research should expand on this foundation by incorporating a more diverse range of participants, including laypeople and professionals from various fields, to ensure that findings are broadly acceptable. Future studies should also recruit participants from around the globe to ensure that AV traffic behavior is compatible and consistent with ethical perspectives outside of North America. Additionally, examining moral judgments in low—and high-stakes scenarios will provide a more comprehensive understanding of traffic morality. Addressing the phenomenon of moral luck and its impact on evaluations will be crucial for refining the ADC model’s application to AVs.

All in all, this study contributes to the growing body of research aligning AV behavior with human moral standards. By elucidating how moral judgments are formed in everyday traffic situations, we take an essential step toward developing AVs that can navigate the moral landscape of our roads with greater ethical sensitivity and societal acceptance. Our findings advocate for continued interdisciplinary collaboration to ensure that the integration of moral decision-making in AVs is both scientifically grounded and ethically sound.

## Data Availability

The raw data supporting the conclusions of this article will be made available by the authors, without undue reservation.
